# Epirubicin is not Superior to Doxorubicin in the Treatment of Advanced Soft Tissue Sarcomas.The Experience of the EORTC Soft Tissue and Bone Sarcoma Group

**DOI:** 10.1155/S1357714X00000062

**Published:** 2000-03

**Authors:** Ole S. Nielsen, Per Dombernowsky, Henning Mouridsen, Søren Daugaard, Martine Van Glabbeke, Anne Kirkpatrick, Jaap Verweij

**Affiliations:** 1 Centre for Bone and Soft Tissue Sarcomas Aarhus University Hospital Aarhus Denmark; 2 Department of Oncology Copenhagen University Hospital Herlev Denmark; 3 The Finsen Center Rigshospitalet Copenhagen Denmark; 4 The Laboratory Center Rigshospitalet Copenhagen Denmark; 5 EORTC Data Centre Brussels Belgium and Department of Medical Oncology The Netherlands; 6 Rotterdam Cancer Institute (Daniel den Hoed Kliniek) and University Hospital Rotterdam The Netherlands

## Abstract

*Purpose.* Doxorubicin (dox) still appears to be one of the most active drugs in the treatment of soft tissue sarcomas. However, treatment duration is limited due to cumulative cardiotoxicity. A number of small studies from single institutions have suggested activity of other analogues. In two studies the EORTC STBSG tested whether epirubicin (epi) is an alternative to standard dose dox in the treatment of chemonaive patients with advanced soft tissue sarcoma. The present report gives the final results of these studies.

*Patients/Methods.* In the first study 210 patients were randomized to receive either dox or epi both at a dose of 75 mg/m^2^ given as bolus injection at 3-week intervals. In the second study 334 patients were randomized to dox 75 mg/m^2^, epi 150 mg/m^2^ or epi 50 mg/m^2^ days 1–3, all given as bolus injection at 3-week intervals.

*Results.* In the first study no differences in median survival and duration of response were found. Of 167 evaluable patients the response rate was slightly in favour of dox (23% vs 18%) but at the expense of more toxicity.These data could suggest that increasing the epi dose may lead to a greater antineoplastic effect with acceptable toxicity. In the second study 15% of 314 evaluable patients had an objective tumour response. There were no differences between the three groups with regard to response rate, progression-free and overall survival, but both dose schedules of epi were more myelotoxic than dox.

*Conclusion.* Regardless of schedule and dose, epi is not superior to dox in the treatment of patients with advanced soft tissue sarcomas. In addition, the results illustrate that the data from small studies of single institutions should always be confirmed by large multi-institutional studies before being taken for granted.


*Sarcoma* (2000) **4**, 31–35

ORIGINAL ARTICLE

### Epirubicin is not superior to doxorubicin in the treatment of advanced soft tissue sarcomas. The experience of the EORTC soft tissue and bone sarcoma group

OLE S. NIELSEN,[1] PER DOMBERNOWSKY,[2] HENNING MOURIDSEN,[3] SØREN DAUGAARD,[4] MARTINE VAN GLABBEKE,[5] ANNE KIRKPATRICK[5] & JAAP VERWEIJ[6]

[1]*Centre for Bone and Soft Tissue Sarcomas, Aarhus University Hospital, Aarhus, Denmark,* [2]*Department of Oncology, Copenhagen University Hospital, Herlev, Denmark,* [3]*The Finsen Center, Rigshospitalet, Copenhagen, Denmark,* [4]*The Laboratory Center, Rigshospitalet, Copenhagen, Denmark,* [5]*EORTC Data Centre, Brussels, Belgium and Department of Medical Oncology,* [6]*Rotterdam Cancer Institute (Daniel den Hoed Kliniek) and University Hospital, Rotterdam, The Netherlands*

Presented in part at the 4th Meeting of the Connective Tumour Oncology Society in Vancouver, 1998.

**Abstract**

*Purpose.* Doxorubicin (dox) still appears to be one of the most active drugs in the treatment of soft tissue sarcomas. However, treatment duration is limited due to cumulative cardiotoxicity. A number of small studies from single institutions have suggested activity of other analogues. In two studies the EORTC STBSG tested whether epirubicin (epi) is an alternative to standard dose dox in the treatment of chemonaive patients with advanced soft tissue sarcoma. The present report gives the final results of these studies.

*Patients/Methods.* In the first study 210 patients were randomized to receive either dox or epi both at a dose of 75 mg/m$^2$ given as bolus injection at 3-week intervals. In the second study 334 patients were randomized to dox 75 mg/m$^2$, epi 150 mg/m$^2$ or epi 50 mg/m$^2$ days 1–3, all given as bolus injection at 3-week intervals.

*Results.* In the first study no differences in median survival and duration of response were found. Of 167 evaluable patients the response rate was slightly in favour of dox (23% vs 18%) but at the expense of more toxicity. These data could suggest that increasing the epi dose may lead to a greater antineoplastic effect with acceptable toxicity. In the second study 15% of 314 evaluable patients had an objective tumour response. There were no differences between the three groups with regard to response rate, progression-free and overall survival, but both dose schedules of epi were more myelotoxic than dox.

*Conclusion.* Regardless of schedule and dose, epi is not superior to dox in the treatment of patients with advanced soft tissue sarcomas. In addition, the results illustrate that the data from small studies of single institutions should always be confirmed by large multi-institutional studies before being taken for granted.

### Introduction

Chemotherapy has been extensively studied in soft tissue sarcomas.[1,2] Unfortunately, their responsiveness to chemotherapy has been disappointingly low. Doxorubicin still appears to be one of the most active drugs.[1] Numerous patients, both non-pretreated and pretreated, have received doxorubicin as a single agent with a reported maximum response rate of about 25%.[1,3–5] Presently, the EORTC Soft Tissue and Bone Sarcoma Group (STBSG) considers single agent doxorubicin as the standard treatment for advanced soft tissue sarcomas. However, doxorubicin treatment is associated with cardiotoxicity, and unfortunately none of the tested anthracycline analogs has shown superiority or comparability to doxorubicin in terms of therapeutic activity with less toxicity.[6–8]

In view of this, the EORTC STBSG has, in two consecutive studies, tested whether epirubicin, which is considered to be less cardiotoxic than doxorubicin,[9] could be an alternative to standard dose doxorubicin in the treatment of chemonaive patients with advanced soft tissue sarcoma. The data from these studies have been published previously, but the present report gives the overall and final results of these studies[10,11] and summarizes the literature on epirubicin in soft tissue sarcomas.

### Patients and methods

Patients and methods have been described in detail in previous publications[10,11] and will only be summarized in the present paper. The studies were

Correspondence to: O. S. Nielsen, Department of Oncology, Aarhus University Hospital, Nörrebrogade 44, DK-8000 Aarhus C, Denmark. Fax: +45 89492550; E-mail: osn@oncology.dk.

1357-714X print/1369-1643 online/00/010031-05 © 2000 Taylor & Francis Ltd conducted in adult chemonaive patients with histologically proven advanced soft tissue sarcomas. Other eligibility criteria included: no history of *significant* cardiovascular disease, no prior malignant tumour, no CNS metastases, presence of measurable lesions not previously irradiated and adequate hepatic, renal and bone marrow functions at entry. Informed consent was obtained from all patients according to local and/or national rules.

The studies aimed to compare time to progression, duration of survival, response rate and response duration, as well as acute and chronic toxicity. In the first study patients were randomized to receive an iv bolus injection of either doxorubicin or epirubicin both at a dose of 75 mg/m$^2$ every 3 weeks, whereas in the second the patients were randomized to receive an iv bolus injection of doxorubicin 75 mg/m$^2$ or epirubicin at a dose of either 150 mg/m$^2$ as a single iv bolus injection or 3 iv bolus injections of 50 mg/m$^2$ on days 1–3, all repeated every 3 weeks. The evaluation of toxicity was done according to the recommendation made by WHO for grading of acute and subacute toxic effects, and in both studies doses were modified according to liver and bone marrow functions. At least two cycles were given, and the maximal accepted cumulative dose was 550 mg/m$^2$ for doxorubicin and 1000 mg/m$^2$ for epirubicin. However, in case of CR it was left to the discretion of the local investigator to continue to a higher cumulative dosage.

Evaluation prior to treatment included history and clinical examination, performance status, tumour measurements, haematology, blood chemistries, plain chest radiograph, ECG, appropriate scans and/or radiographs for tumour measurements, and cardiac ejection fraction. Blood counts were performed weekly during treatment for the initial two treatment cycles. At follow-up clinical examination blood counts and chemistries were performed before every cycle. All baseline investigations were reported every second course.

Patients were considered assessable for response if they had received at least two cycles of chemotherapy. Response was defined according to the WHO criteria. All cases were reviewed by an external reviewer. Exact 95% confidence intervals for proportions were calculated for response rates. Duration of response, progression-free and overall survival were estimated by use of the Kaplan–Meyer method.[12] The log-rank test was used for comparison between survival curves.[13] Calculation of study power was done according to standards of the EORTC STBSG.[11]

### Results

### *Patient characteristics*

In both studies the covariates age, sex, performance status, histological grades, sites of involvement, and prior treatment were evenly distributed among the treatment groups. Patient characteristics are given in Table 1. In the first study 210 patients were entered by 18 institutions. A total of 28 patients were considered ineligible due to inadequate histology ($n$=16), previous chemotherapy ($n$=3), insufficient performance status ($n$=2), non-measurable lesions ($n$=4) and others ($n$=3). Of the 182 eligible patients, 15 were not evaluable (Table 1). In the second study a total of 334 patients from 34 centers were included. Fifteen patients were considered as ineligible for the trial for the following reasons: inadequate histology ($n$=8), no target lesion ($n$=1), concurrent disease ($n$=2), age >70 years ($n$=1), performance status >2 ($n$=1), prior breast cancer ($n$=1) and prior chemotherapy ($n$=1). An additional 5 patients were lost during follow-up. In total 20 patients were excluded from the analysis, which consequently was based on 314 patients (Table 1).

A central histopathology review was performed in 89% and 83% of the patients, respectively. Leiomyosarcomas contributed 32% and 40% whereas malignant fibrous histiocytoma contributed 21% and 10%, respectively. The histopathological types were equally distributed among the treatment groups (data not shown). Prior radiotherapy was given to 31% and 22% of the patients, respectively.

### *Treatment compliance*

In neither of the studies did the treatment compliance differ among the treatment groups. In the first study the patients received a median of 5 cycles (1–23), and the median total dose in the 2 groups was 338 mg/m$^2$ (75–916) of doxorubicin and 363 mg/m$^2$ of epirubicin, respectively. In the second study the patients received a median of 4 cycles (0–11), and the median total dose and the relative dose intensity as computed according to the Hryniuk method[13] were for doxorubicin 299 mg/m$^2$ (50–599) and 97%, for 1-day epirubicin 592 mg/m$^2$ (131–1343) and 94%, and for 3-day epirubicin 481 mg/m$^2$ (50–1105) and 92%, respectively.

### *Toxicity*

When given in equimolar doses doxorubicin caused more haematological toxicity (Table 2) and alopecia

**Table 1.** *Patients' Characteristics*

| Characteristics | Study No. 1 | Study No. 2 |
|---|---|---|
| Registered patients | 210 | 334 |
| Ineligible patients | 28 | 15 |
| Insufficient data | 15 | 5 |
| Included patients | 167 | 314 |
| Median age, years | 54 (18–80) | 52 (19–70) |
| Male/females | 87/80 | 154/160 |
| Histological grade | | |
| 1 | 22 (21%) | 65 (21%) |
| 2 | 38 (37%) | 124 (39%) |
| 3 | 44 (42%) | 125 (40%) |

**Table 2.** *Worst WHO grades 3+4 haematological toxicities across all cycles in the different treatment regimens (% of patients)**

| Treatment | Leucopenia (%) | Neutropenia (%) | Thrombocytopenia (%) |
|---|---|---|---|
| Dox 75 mg/m$^2$ | 29 | 43 | 4 |
| Epi 75 mg/m$^2$ | 4 | 3 | 0 |
| Dox 75 mg/m$^2$ | 38 | 51 | 2 |
| Epi 150 mg/m$^2$ | 63 | 73 | 14 |
| Epi 3×50 mg/m$^2$ | 75 | 77 | 18 |

*See original publications for a detailed description of toxicities.[10,11]

as compared with epirubicin, whereas there was no difference between the other toxicities registered (data not shown). Increasing the dose of epirubicin resulted in more severe toxicity with both the 1-day and the 3-day regimen as compared with doxorubicin in terms of haematological toxicity (Table 2) and rate of infection. Apart from these toxicities very few grade 3 and grade 4 toxicities were observed, and no significant differences were observed between the other toxicities (data not shown). In the 3-day epirubicin group three toxic deaths were reported: two patients died of neutropenic infection and one due to cardiotoxicity. One patient died of cardiotoxicity after 8 cycles of doxorubicin.

*Response*

The progression-free survival was similar with the three treatment schedules studied (Fig. 1). Neither did the overall survival differ between the treatment schedules (Fig. 2). The survival curve characteristics are given in Table 3.

The response data are shown in Table 4. The 'progression' category includes early progressions as well as early deaths due to malignant disease. In the first study the overall response rate was 25% in the group receiving doxorubicin and 18% in the group treated with epirubicin, but the difference was not

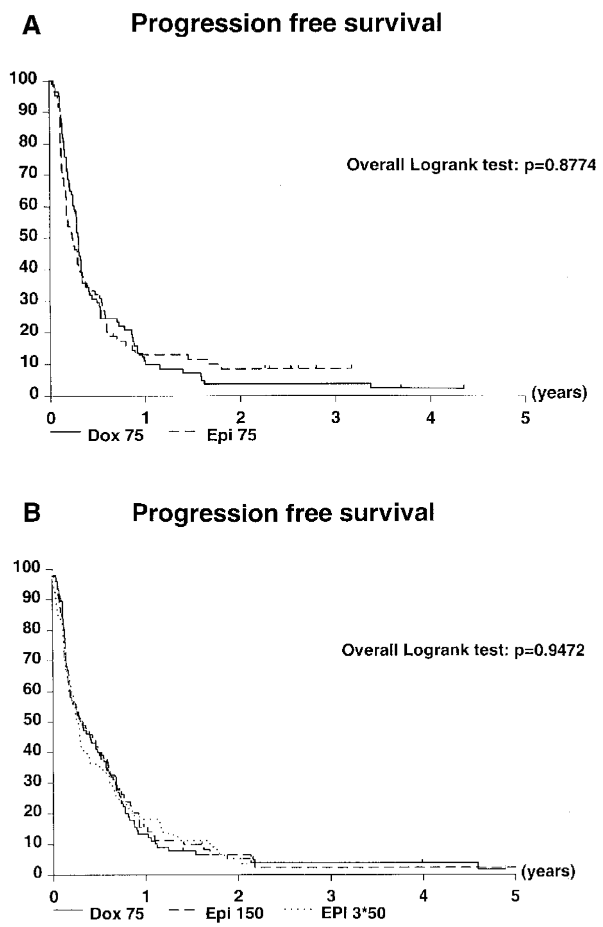

**Figure 1.** *Actuarial estimate of progression-free survival of patients with soft tissue sarcoma treated with A: Doxorubicin 75 mg/m$^2$ day 1 or epirubicin 75 mg/m$^2$ day 1, and B: Doxorubicin 75 mg/m$^2$ day 1, epirubicin 150 mg/m$^2$ day 1 or epirubicin 50 mg/m$^2$ days 1, 2 and 3 every 3 weeks.*

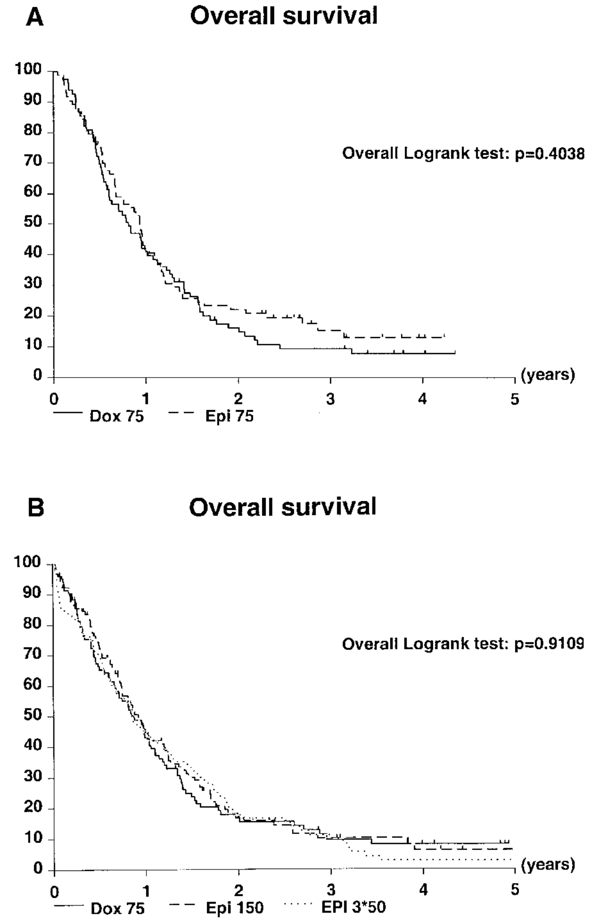

**Figure 2.** *Actuarial estimate of overall survival of patients with soft tissue sarcoma treated with A: Doxorubicin 75 mg/m$^2$ day 1 or epirubicin 75 mg/m$^2$ day 1, and B: Doxorubicin 75 mg/m$^2$ day 1, epirubicin 150 mg/m$^2$ day 1 or epirubicin 50 mg/m$^2$ days 1, 2 and 3 every 3 weeks.*

**Table 3.** *Progression-free survival (PFS) and overall survival (OS) characteristics**

| Treatment | 1-year PFS (%) | Median PFS (weeks) | 1-year OS (%) | Median OS (weeks) |
|---|---|---|---|---|
| Dox 75 mg/m$^2$ | 11 | 16 | 41 | 41 |
| Epi 75 mg/m$^2$ | 14 | 12 | 41 | 48 |
| Dox 75 mg/m$^2$ | 13 | 17 | 45 | 45 |
| Epi 150 mg/m$^2$ | 15 | 16 | 43 | 48 |
| Epi 3×50 mg/m$^2$ | 18 | 14 | 45 | 45 |

*See original publications for a detailed description of toxicities.[10,11]

significant. Neither was there any difference in response rates among the three groups of the second study. The overall response rates were 14% in the doxorubicin group, 15% in the 1-day epirubicin group, and 14% in the 3-day epirubicin group. The data in Table 4 may suggest that the response rates are lower in the second study as compared with those of the first study.

### Discussion

The present studies on advanced soft tissue sarcomas showed that equimolar doses of doxorubicin and epirubicin produced response rates favouring doxorubicin (although not statistically significant) but at the cost of more toxicity. Despite a significant increase of both haematological and non-haematological acute side-effects, none of the two tested high-dose epirubicin schedules demonstrated any superior outcome compared with standard dose doxorubicin. Similarly, both the progression-free and overall survival were similar to those obtained in other trials performed by the EORTC STBSG in comparable patients. However, the response rates obtained in the second of the presented studies were disappointing and lower than that of the first study (Table 4). A similar trend of decreasing response rates has been found in other studies.[1,7,9,11,15] A difference in dose intensity between the two studies was not the reason for the poor response rate. Neither was there a difference in patient selection (prognostic factors) between the two groups. In the second study the number of leiomyosarcomas was slightly higher than that of the first study—a difference that may partly explain the lower response rates. Both studies were also subject to the same response review system, making it unlikely that differences in responses reported offers an explanation. We are presently analysing possible explanations for these low response rates. On the other hand, although the response rates were lower in the second study, the progression rates remained the same (Table 3).

Treatment duration with doxorubicin is limited because of cardiotoxicity associated with cumulative dose. It is therefore important to test anthracycline analogs with potentially less toxicity and equal or better activity. However, at present only few analogs have been evaluated and primarily in small studies from single institutions with limited number of patients.[6–8,16–18] Some of these studies indicated that epirubicin may be active in soft tissue sarcomas, whereas carminomycin, mitoxantrone as well as other analogs were shown to be inactive. Some studies have indicated that epirubicin may be very active as a part of combination chemotherapy regimens but whether it's a better alternative than doxorubicin has not been demonstrated in these studies.[20,21] The present results on epirubicin may illustrate that the data from such small studies of single institutions should always be confirmed by large multi-institutional studies before being taken for granted. Pegylated liposomal doxorubicin (caelyx) represents a novel formulation to deliver doxorubicin, which may increase tumour effect with less toxicity. In a phase 2 second line study caelyx did not show activity in soft tissue sarcomas.[19] In the EORTC STBSG we recently completed a randomized phase 2 study investigating caelyx and doxorubicin given as first line treatment in soft tissue sarcomas, but at present data analysis is still ongoing.[22] Finally, the possibility of ameliorating anthracycline cardiotoxicity by use of compounds like dexrazoxane is presently under investigation.[23]

In conclusion, regardless of schedule and dose epirubicin is not superior to doxorubicin in the treatment of patients with advanced soft tissue sarcomas.

**Table 4.** *Response to treatment**

| Treatment | CR (%) | PR (%) | NC (%) | PD (5) | NE (%) |
|---|---|---|---|---|---|
| Dox 75 mg/m$^2$ | 7 | 18 | 45 | 30 | — |
| Epi 75 mg/m$^2$ | 5 | 13 | 40 | 42 | — |
| Dox 75 mg/m$^2$ | 2 | 12 | 50 | 36 | — |
| Epi 150 mg/m$^2$ | 3 | 12 | 43 | 41 | — |
| Epi 3×50 mg/m$^2$ | 3 | 11 | 39 | 43 | 5 |

*CR, complete response; PR, partial response; NC, no change; PD, progressive disease; NE, not evaluable.
See original publications for a detailed description of toxicities.[10,11]

### References

1 Verweij J, Mouridsen HT, Nielsen OS, *et al.* The present state of the art in chemotherapy for soft tissue sarcomas in adults: the EORTC point of view. *Crit Rev Oncol/Hematol* 1995;20:193–201.

2 Suit HD. Tumours of the connective and supporting tissues. *Radioth Oncol* 1995;34:93–104.

3 O'Bryan RM, Luce JK, Talley RW. Phase II evaluation of adriamycin in human neoplasm. *Cancer* 1973;32:1–8.

4 Borden EC, Amato DA, Rosenbaum CH. Randomized comparison of three adriamycin regimens for metastatic soft tissue sarcomas. *J Clin Oncol* 1987;5:840–50.

5 Blackledge G, van Oosterom AT, Mouridsen HT, *et al.* Doxorubicin in relapsed soft tissue sarcoma: Justification of phase II evaluation of new drugs in this disease: An EORTC soft tissue and bone sarcoma group study. *Eur J Cancer Clin Oncol* 1990;26:139–41.

6 Suit HD, van Groeningen CJ, Mankin HJ, Rosenberg AE. Sarcoma of the soft tissues: In: Peckham M, Pinedo H, Veronesi U, eds. *Oxford Textbook of Oncology.* Oxford: Oxford University Press, 1995:1917–39.

7 Bramwell VHC, Mouridsen HT, Mulder JH, *et al.* Carminomycin vs. adriamycin in advanced soft tissue sarcomas: an EORTC randomized phase II study. *Eur J Cancer Clin Oncol* 1983;19:1097–104.

8 Bull FE, von Hoff DD, Balcerak SP, Stephens RL, Panettiere FJ. Phase II trial of mitoxantrone in advanced sarcomas: a Southwest Oncology Group study. *Cancer Treat Reports* 1985;69:231–3.

9 Launchbury AP, Habboubit N. Epirubicin and doxorubicin: a comparison of their characteristics, therapeutic activity and toxicity. *Cancer Treatm Rev* 1993;19:197–228.

10 Mouridsen HT, Bastholt L, Somers R, *et al.* Adriamycin versus epirubicin in advanced soft tissue sarcomas. A randomized phase II/phase III study of the EORTC soft tissue and bone sarcoma group. *Eur J Cancer Clin Oncol* 1987;23:1477–83.

11 Nielsen OS, Dombernowsky P, Mouridsen H, *et al.* High-dose epirubicin is not an alternative to standard-dose doxorubicin in the treatment of advanced soft tissue sarcomas. A study of the EORTC soft tissue and bone sarcoma group. *Br J Cancer* 1998;78:1634–9.

12 Kaplan E, Meier P. Non-parametric estimation from incomplete observations. *J Am Stat Assoc* 1958;53:457–81.

13 Peto R, Pike Mc, Armitage P, *et al.* Design and analysis of clinical trials requiring prolonged observation of each patient. *Br J Cancer* 1977;35:1–39.

14 Hryniuk W, Bush H. The importance of dose intensity in chemotherapy of metastatic breast cancer. *J Clin Oncol* 1984;2:1281–8.

15 Santoro A, Tursz T, Mouridsen HT, *et al.* Doxorubicin versus CYVADIC versus doxorubicin plus ifosfamide in first-line treatment of advanced soft tissue sarcomas: A randomized study of the EORTC soft tissue and bone sarcoma group. *J Clin Oncol* 1995;13:1537–45.

16 Chevalier B, Monteuquet PH, Fachini T, *et al.* Phase II study of epirubicin in advanced soft tissue sarcomas. *Proc Am Soc Clin Oncol* 1988;8:323.

17 Jelic S, Vuletic L, Milanovic N, Tomasevic Z, Kovcin V. High-dose epirubicin–cisplatin chemotherapy for advanced soft tissue sarcoma. *Tumori* 1990;76:467–71.

18 Plosker GL, Faulds D. Epirubicin. A review of its pharmacodynamic and pharmacokinetic properties, and therapeutic use in cancer chemotherapy. *Drugs* 1993;45:788–856.

19 Garcia AA, Kempf RA, Rogers M, Muggia FM. A phase II study of Doxil (liposomal doxorubicin): Lack of activity in poor prognosis soft tissue sarcomas. *Annals Oncol* 1998;9:1131–3.

20 Reichardt P, Tilgner J, Hohenberger P, Dorken B. Dose-intensive chemotherapy with ifosfamide, epirubicin, and filgrastim for adult patients with metastatic or locally advanced soft tissue sarcoma: a phase II study. *J Clin Oncol* 1998;16:1438–43.

21 Palumbo R, Neumaier C, Cosso M, *et al.* Dose intensive first-line chemotherapy with epirubicin and continuous infusion ifosfamide in adult patients with advanced soft tissue sarcomas: a phase II study. *Eur J Cancer* 1999;35:66–72.

22 Judson I, Radford J, Blay J-Y, *et al.* A randomised phase II trial of caelyx/doxil versus doxorubicin in advanced or metastatic soft tissue sarcomas (STS)—An EORTC Soft Tissue and Bone Sarcoma Group (STBSG) trial. *Proc of ASCO* 1999;18:541a.

23 Wexler LH. Ameliorating anthracycline cardiotoxicity in children with cancer: Clinical trials with dexrazoxane. *Semin Oncol* 1998;25(suppl 10):86–92.
